# Use of Bead-Based Serologic Assay to Evaluate Chikungunya Virus Epidemic, Haiti

**DOI:** 10.3201/eid2406.171447

**Published:** 2018-06

**Authors:** Eric W. Rogier, Delynn M. Moss, Kimberly E. Mace, Michelle Chang, Samuel E. Jean, Stevan M. Bullard, Patrick J. Lammie, Jean Frantz Lemoine, Venkatachalam Udhayakumar

**Affiliations:** Centers for Disease Control and Prevention, Atlanta, Georgia, USA (E.W. Rogier, D.M. Moss, K.E. Mace, M. Chang, S.M. Bullard, P.J. Lammie, V. Udhayakumar):; Population Services International/Organisation Haïtienne de Marketing Social pour la Santé, Port-au-Prince, Haiti (S.E. Jean);; Programme National de Contrôle de la Malaria/MSPP, Port-au-Prince (J.F. Lemoine)

**Keywords:** immunoassay, serology, *Aedes* *aegypti*, Albopictus, mosquitoes, arbovirus, chikungunya, CHIKV, Haiti, viruses, vector-borne infections

## Abstract

The index case of chikungunya virus (CHIKV) in Haiti was reported during early 2014; the vector, the pervasive *Aedes aegypti* mosquito, promoted rapid spread throughout the country. During December 2014–February 2015, we collected blood samples from 4,438 persons at 154 sites (62 urban, 92 rural) throughout Haiti and measured CHIKV IgG by using a multiplex bead assay. Overall CHIKV seroprevalence was 57.9%; differences between rural (mean 44.9%) and urban (mean 78.4%) areas were pronounced. Logistic modeling identified the urban environment as a strong predictor of CHIKV exposure (adjusted odds ratio 3.34, 95% CI 2.38–4.69), and geographic elevation provided a strong negative correlation. We observed no correlation between age and antibody positivity or titer. Our findings demonstrated through serologic testing the recent and rapid dissemination of the arbovirus throughout the country. These results show the utility of serologic data to conduct epidemiologic studies of quickly spreading mosquitoborne arboviruses.

Chikungunya virus (CHIKV) is an arbovirus, transmitted by *Aedes aegypti* and *Ae*. *albopictus* mosquitoes, that can cause transient but debilitating disease in humans. The World Health Organization reported the first cases of CHIKV on the island nation of Haiti in April 2014; by June 2014, a total of 6,318 cases had been reported there and in 16 other countries or territories in the Caribbean and South America; 103,018 suspected cases were reported (*1*)*.* Additional evidence that CHIKV was introduced into Haiti in 2014 came from evaluation of a longitudinal cohort of children in the coastal town of Leogane during 2011–2014. Before 2014, these children tested negative for CHIKV antibodies, but samples collected in 2014 showed CHIKV IgG; 78.9% of all children seroconverted within the span of 1 year (*2*).

Confirmation of active CHIKV infection is accomplished through reverse transcription PCR or detection of CHIKV IgM (*3**,**4*)*.* Although confirming infection aids in determining the causative agent of symptoms, only supportive care is currently available for chikungunya, because CHIKV-specific antiviral drugs have not been identified (*5*)*.* Furthermore, using these assays would require persons to have been sampled during active or recent viremia, whereas CHIKV IgG could persist for longer periods of time (*4**,**6*). We present data from a nationwide survey in Haiti in which we used a bead-based serologic assay to determine the overall presence of CHIKV IgG, which provides evidence of past and current exposure.

## Materials and Methods

### Study Population

Our group, which includes Population Services International (PSI), conducted a survey to evaluate malaria prevalence in Haiti during December 2014–February 2015 as part of the malaria control activities supported by the Global Fund­ (https://www.theglobalfund.org/en/malaria/). In addition to planned multiplex serologic testing for malaria, we chose antigens for nonmalarial febrile diseases before starting data collection; these tests were approved by the Haitian Ministry of Health. The Institut Haïtien de Statistiques et d’Informatique (http://www.ihsi.ht/) had previously subdivided the nation into 12,000 enumeration areas (sections d’énumération, SDEs) on the basis of population density; we chose 154 of these SDEs for this survey through proportional sampling of predicted malaria risk strata within the country, as had been determined by predictive modeling (*7*). The SDEs were classified as urban if they were within the administrative boundary of any of the 140 municipal cities in Haiti and were otherwise considered rural. Field teams randomly selected 20 households within each SDE; all members of selected households were offered participation. Following verbal consent by the participant (or parents or guardian if <15 years of age), blood was collected by fingerprick on Whatman 903 Protein Saver cards (GE Healthcare, Marlborough, MA, USA), dried overnight, and individually stored in plastic bags with desiccant. We assigned samples unique identification numbers that were not traceable to the individual persons. The study protocol was approved by the Haitian Ministry of Health and approved as a non-research activity by the US Centers for Disease Control and Prevention (CDC; Center for Global Health determination #2015-04). 

A total of 4,438 participants, 1–99 years of age, were included in the survey; the median number of persons sampled per site was 30. During sample collection, we logged global positioning system coordinates for each SDE; we later obtained elevation above sea level in meters by using a digital elevation map of Haiti that was accurate within 1 m.

### Antigen Coupling to Microbeads and Direct Comparison with Anti-CHIKV IgG ELISA

The CHIKV IgG bead assay was designed by CDC laboratories (*2*), and the assay was conducted at CDC laboratories (Atlanta, GA, USA). Carboxyl groups on the surface of specifically classified spectral polystyrene microspheres (BioPlex microbeads; Bio-Rad, Hercules, CA, USA) were converted to reactive esters by using the 1-ethyl-3-(3-dimethylaminopropyl) carbodiimide method (Calbiochem, Woburn, MA, USA). The recombinant CHIKV wild-type and mutant (A226V) envelope 1 (E1) antigens (CTK Biotech, San Diego, CA, USA), 7.5 μg each, were covalently linked to 1 mL (12.5 × 10^6^ microbeads) of activated microspheres by amide bonds by using phosphate-buffered saline (pH 7.2). To confirm the coupling reaction, the serum we tested was previously found to be highly reactive to the antigens; these showed high median fluorescence intensity (MFI) minus background (MFI-bg) values, indicating appropriate antigen coupling to the microbeads. A seropositivity MFI-bg cutoff value for the antigen-coupled microbeads was determined by using 86 serum specimens from adults from the United States who had not traveled internationally. Of the 86 specimens, 2 outliers had MFI-bg readings >2 SDs above the mean and were eliminated from the analysis as influential outliers. We defined the lower limit for seropositivity to IgG as 594 MFI-bg, which was the fluorescence intensity 3 SD above the mean MFI for the remaining 84 samples.

We evaluated the sensitivity and specificity of the bead assay in comparison to the anti-CHIKV IgG ELISA protocol used by CDC’s Division of Vector-Borne Diseases, National Center for Emerging and Zoonotic Infectious Diseases (*2**–**4*). For the ELISA, the viral antigen came from the brain of a suckling mouse and was captured with a monoclonal antibody. Any IgG from a test serum that reacted with this antigen was probed by using goat anti-human IgG linked to alkaline phosphatase. We developed color by using disodium p-nitrophenyl phosphate and read at 405 nm. We subtracted optical density (OD) of a blank well containing sample diluent from the test serum to report a final value of OD minus background (OD-bg). We tested serum samples (n = 50) from CHIKV endemic and nonendemic regions by using ELISA and the bead assay; test comparisons for continuous data are shown in Technical Appendix Figure 1, panel A and binary IgG positive/IgG negative data in Technical Appendix Figure 1, panel B. When we used ELISA as the standard assay, the sensitivity of the bead assay was estimated to be 90% and specificity was 85%. The bead assay signal for anti-CHIKV IgG in Haitians was basically nonexistent before CHIKV introduction (Technical Appendix Figure 2).

### Dried Blood Spot Elution and Data Acquisition

Elution of whole blood from dried blood spots (DBSs), detection of the IgG bound to the CHIKV antigen-coupled microbeads, and data acquisition by the multiplex bead assay have been described (*8*)*.* In brief, we took a 6-mm circular punch corresponding to 14 μL whole blood from the center of each DBS for elution. Samples were shaken overnight at room temperature in 140 μL protein elution buffer containing PBS (pH 7.2), 0.05% Tween-20, and 0.05% sodium azide. Samples were then stored at 4°C until analysis. Elution from blood spots provided an initial 1:10 dilution of whole blood, and samples were further diluted 1:40 in sample diluent for a final whole blood dilution of 1:400, corresponding to a serum dilution of ≈1:800 on the basis of the assumption of 50% hematocrit in whole blood. We diluted samples in a blocking buffer (sample diluent) containing 0.5% polyvinyl alcohol (Sigma, St. Louis, MO, USA), 0.8% polyvinylpyrrolidine (Sigma), 0.1% casein (ThermoFisher Scientific, Waltham, MA, USA), 0.5% bovine serum albumin (Millipore, Burlington, MA, USA), 0.3% Tween-20, 0.1% sodium azide, and 0.01% *E. coli* extract to prevent nonspecific binding. Assay reagent diluent (Buffer C) consisted of PBS-Tween (ThermoFisher Scientific, Waltham, MA, USA) plus 0.5% bovine serum albumin and 0.02% sodium azide. We prewetted filter bottom plates (Multiscreen 1.2 μmol/L, Millipore) with PBS-Tween, added 1,500 microbeads/classification each well, and incubated with sample in duplicate for 1.5 h under gentle shaking. We then added secondary antibodies tagged with biotin (1:500 anti-human IgG_1–3_; Southern Biotech, Birmingham, AL; 1:2,500 anti-human IgG_4_; Sigma) and incubated for 45 min. Next, we added streptavidin-phycoerythrin (1:200; Invitrogen, Carlsbad, CA, USA) and incubated for 30 min. Plates had a final wash incubation with Buffer C for 30 min and were read on a Bio-Plex 200 instrument by generating the median fluorescence signal for 50 microbeads/analyte. We calculated the mean from duplicate wells, each with an MFI (1 – 32,766 channels) by using Bio-Plex Manager 6.1 software (Bio-Rad). We subtracted background from a DBS blank from all sample MFI values to give a final MFI-bg value that we used for analysis.

### Statistical Methods

We used the Mann-Whitney rank sum test to determine differences between groups for continuous variables and the z-test to determine the significance of differences between 2 groups for proportions. We considered p<0.05 statistically significant. We modeled the relationship between IgG against chikungunya E1 antigen and urban environment, elevation, and age by using logistic regression (GENMOD procedure in SAS version 9.4; SAS Institute, Cary, NC, USA) and reported 95% Wald CIs with a null hypothesis of β*_x_* = 0. The final logistic regression model was written as logit P(chikpos = 1) = β_0_ + β_1_(urban setting) + β_2_(elevation[m]) + β_3_(age[years]). We used generalized estimating equations to account for clustering at the SDE level when obtaining p values and 95% CIs.

## Results

### Prevalence of IgG Responses to CHIKV

**Figure 1:**
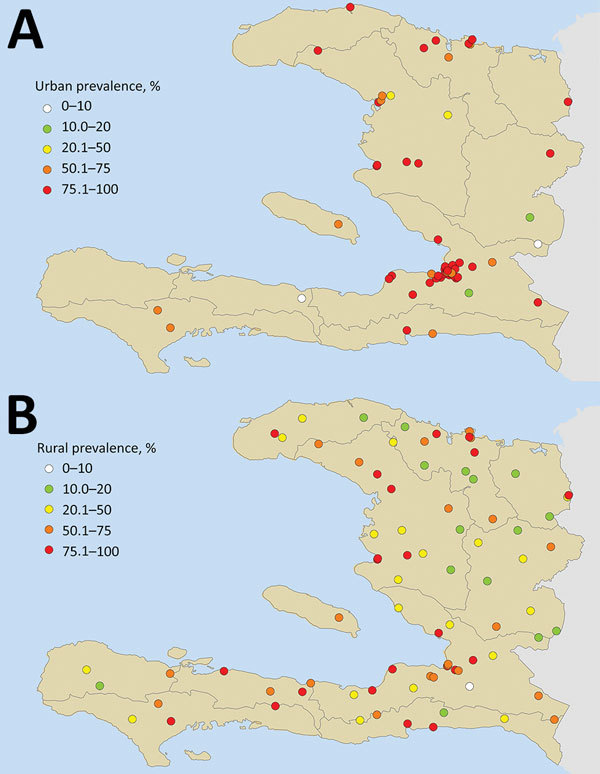
Urban (A) and rural (B) serosurvey sampling sites for chikungunya prevalence, Haiti, December 2014–February 2015. Geolocated point seroprevalence is shown as the percentage of the sampled population positive for chikungunya IgG.

The overall CHIKV antibody seroprevalence was 57.9% (2,570/4,438 persons). However, rates of seropositivity were highly variable: 78.4% (1,350/1,722) prevalence in urban areas (range 20%–100%) and 44.9% (1,220/2,716) in rural areas (range 8%–100%) (Figure 1). Many of the rural areas sampled were located inland, away from the coast and at higher elevations. In contrast, many of the urban sampling sites were located nearer the coast at lower elevations. Median IgG responses in urban areas (MFI-bg 1,856) were significantly higher (p<0.001) than median IgG responses in the rural areas (MFI-bg 298), and median elevation above sea level in the urban areas (38 m) was significantly lower than the median elevation in the rural areas (228 m; p<0.001).

### Relationship between Seroprevalence and IgG Titer with Elevation

**Figure 2:**
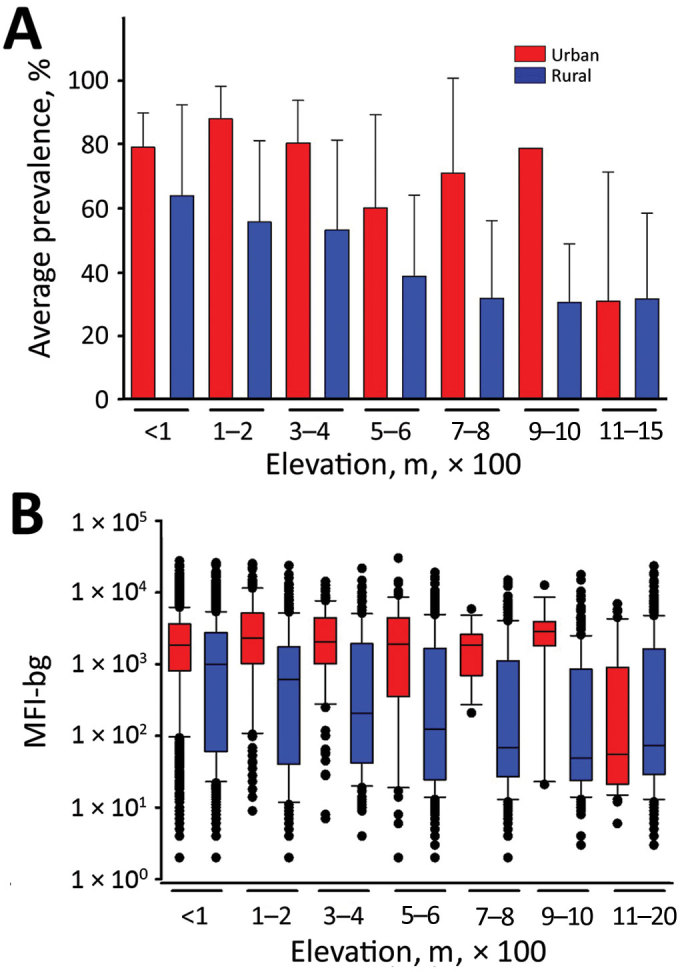
Seroprevalence and chikungunya IgG levels among persons living in urban and rural areas at different elevations, Haiti, December 2014–February 2015. A) Seroprevalence mean of persons sampled in urban or rural areas at different elevations; error bars indicate SEM. B) Chikungunya IgG median fluorescence intensity minus background signal by urban and rural sampling sites at different elevations. Boxes indicate interquartile ranges; horizontal lines within boxes indicate medians; black dots indicate values <10th or >90th percentiles; error bars indicate 10th and 90th percentiles of data. M, meters; MFI-bg, median fluorescence intensity minus background.

The prevalence of positivity for CHIKV IgG and MFI-bg assay signal (indicating the magnitude of a person’s IgG response), by elevation categories, for urban and rural areas is shown in Figure 2. Modeling to account for the effects of the urban setting, elevation, and age showed a substantial increase in likelihood of a patient’s sample testing CHIKV IgG–positive in an urban setting (adjusted odds ratio of 3.34; Wald *X*^2^ 48.7; 95% CI 2.38–4.69), considering the rural setting as the referent and accounting for the within-subject factor of sampling multiple persons within an SDE. For every 100 m increase in elevation, the log odds of CHIKV IgG positivity decreased 14.8%; the interaction between elevation and the urban setting was not significant (Wald *X*^2^ 0.56; p = 0.45). Seroprevalence estimates remained higher for populations sampled in urban settings <600 m elevation and steadily decreased among increasing elevation of rural communities (Figure 2, panel A). In directly comparing urban versus rural CHIKV IgG seroprevalence for each elevation category, only the difference within the 100–200 m category was found to be significant (p = 0.024), although urban/rural differences for other categories at elevations <600 m approached statistical significance (p = 0.06–0.09). We found no difference in the rate of IgG positivity when comparing urban and rural settings at >600 m (p = 0.29), but this segment only accounted for 9.5% of the study population.

We saw a more striking difference between urban and rural areas when maintaining a continuous scale for antibody titer. At all elevation categories <600 m, we found a significantly higher IgG titer for persons living in urban versus rural areas (p<0.001 for all elevations), but we found no difference in MFI-bg for persons living at elevations >600 m (p = 0.26) (Figure 2, panel B). Altogether, we found a significantly higher median IgG response (p<0.001) for persons living at elevations <600 m (1,217 MFI-bg) than for persons living at elevations >600 m (66 MFI-bg). When the analysis was restricted to persons living <600 m elevation, modeling for the relationship between IgG titer and elevation showed a sharp decrease of 216 MFI-bg units (F value 47.0; p*<*0.001) in rural settings for every 100 m increase in elevation. In the urban setting, we found a predicted increase in IgG titer for every 100 m increase in elevation, but this increase was not significant (p = 0.12).

### Relationship between Seroprevalence and IgG Titer and Age by Urban and Rural Setting

**Figure 3:**
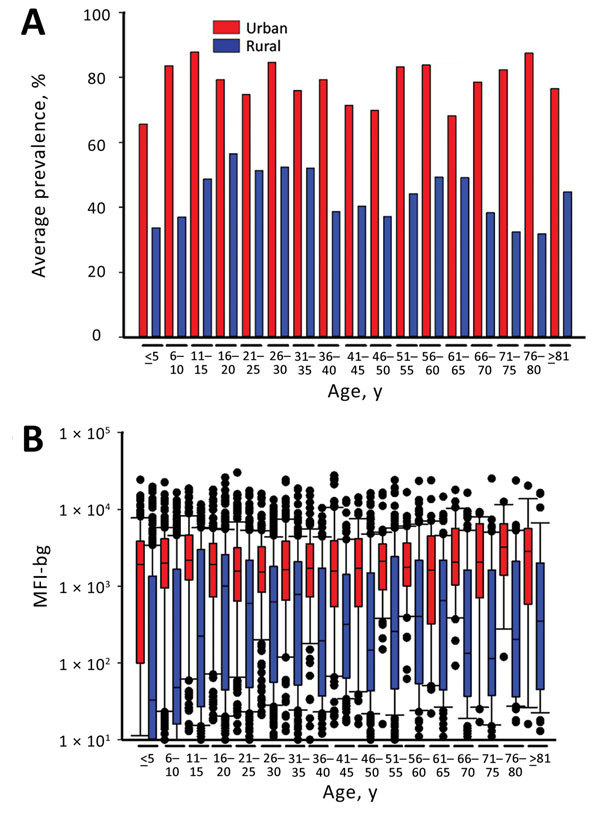
Seroprevalence and chikungunya IgG levels among persons living in urban and rural areas, by age group, Haiti, December 2014–February 2015. A) Mean seroprevalence by urban or rural setting and age category. B) Range of median fluorescence intensity minus background (IgG responses) to chikungunya antigens for the same age categories. Bars indicate interquartile ranges; horizontal lines within bars indicate medians; black dots indicate values >10th or <90th percentiles; error bars indicate 10th and 90th percentiles of data. y, years; MFI-bg, median fluorescence intensity minus background.

Seroprevalence of CHIKV IgG and a plot of median MFI-bg (magnitude of IgG response) by age categories for persons living in urban versus rural areas are shown in Figure 3. These graphs show similar patterns; seroprevalence and MFI-bg responses were consistently higher from persons living in urban versus rural areas, regardless of age. Median MFI-bg was higher in urban areas when compared with rural areas for all age groups (p<0.01 for all categories except age >80 y, p = 0.03) (Figure 3, panel B). By using regression models, we did not find age to be a significant predictor when considering either seroprevalence of CHIKV IgG (p = 0.34) or magnitude of the IgG response (p = 0.19), indicating equivalent probability of lifetime exposure for all Haiti residents, regardless of age. Children 0–10 years of age in rural areas showed exceptionally low median MFI-bg compared with urban children of the same age group.

## Discussion

From this serosurvey, we found 57.9% of the 4,438 residents of Haiti that were tested had IgG responses to CHIKV by early 2015. Although our sample was not nationally representative, we may have underestimated true exposure rates, because 38.8% of persons sampled resided in urban areas, and population estimates for Haiti calculated the percentage of the population in cities at 48.8% (*9*)*.* We found a seroprevalence of 78.4% in urban areas, similar to the 78.9% seroprevalence previously found in the urban area of Leogane in August 2014 (*10*)*.* Considering that the first confirmed cases of CHIKV in Haiti were reported in April 2014 (the same month confirmed cases were seen in the Dominican Republic [*1*]), it is notable that >50% of the population would have evidence of exposure to this arbovirus within 9 months of introduction. The fast spread of CHIKV has been observed before: in the French island of La Reunion in the 2005–2006 outbreak, more than one third of the population was believed to have been exposed, and 63% of the persons living on the island of the Union of the Comoros were exposed to CHIKV during the initial outbreak there (*11**,**12*)*.*

One of our most striking findings was that, even after controlling for age and elevation, persons in urban areas had a >3-fold increase in odds (adjusted odds ratio 3.34) of CHIKV exposure. This result, in combination with the increased likelihood of CHIKV exposure at lower elevation, is directly in line with the consistent observation that the *Aedes* vector house index (HI) is typically higher in these settings. Multiple studies have observed an increase in *Ae. aegypti* vector HI and breeding sites in urban areas when compared with rural locations (*13**–**17*)*.* Previous findings showing increased prevalence of *Ae. aegypti* mosquito colonization in areas of high-density housing and higher water temperature allude to the importance of the urban environment for this vector (*18*). These similar behavioral patterns have recently been observed in Haiti, where *Ae. aegypti* mosquitoes are readily observed in urban and periurban settings and have a propensity for manmade habitats (*19**,**20*)*.* Although *Aedes* sp. mosquito populations have not been mapped by elevation in recent surveys in Haiti, past studies showed substantial decreases in prevalence of hemagglutination inhibition antibodies to dengue virus (as well as to the vector) in moving from low-lying coastal to mountainous inland areas (*21*)*.* We observed a gradual and consistent decrease in seroprevalence to CHIKV IgG for rural areas of increasing elevation >600 m, but this same pattern was less striking for persons sampled from urban areas. This finding may indicate the preservation of *Ae. aegypti* mosquito habitat in urban settings, even at elevations in the hundreds of meters in Haiti; previous entomological work has found limits of distribution of this vector to be largely related to temperature (*22*). 

Persons residing >600 m elevation showed no difference in seroprevalence or CHIKV IgG titers regardless of whether they lived in an urban or rural setting. This finding shows that urban environments in Haiti become less suitable for vector habitat when located at areas of increasing elevation and decreasing temperatures (*23*)*.* Unfortunately, because <10% of our study population resided at elevations >600 m, we were unable to further segregate higher elevation categories. 

A notable limitation to our study is the assumption of persons acquiring CHIKV infection at the geographic location of residence, as this was the location in which persons were sampled. Travel history was not gathered for participants in this study.

In our study, all age groups living in urban areas showed substantially higher MFI-bg signal intensity (IgG responses) to the CHIKV antigens compared with the same age groups living in rural areas. Differences were not seen between IgG prevalence or titer between younger and older age categories, which is atypical for serologic studies of infectious diseases (*2**,**9**,**24**,**25*), because an older age indicates more life-years of potential exposure to an endemic infectious disease and, thus, acquisition of IgG. These serologic findings provide strong evidence for the rapid dissemination of CHIKV by the *Aedes* vector. It appears all age groups in the population were uniformly susceptible to a high rate of transmission, with a preference for urban populations. Of note was the finding that children <10 years of age living in rural areas had lower CHIKV seroprevalence and titers; possible explanations for this include reduced mosquito exposure or transmission efficiency in this setting, or differential behavior patterns among these children as compared to their urban counterparts.

The multiplex bead assay is an excellent serosurvey platform that can simultaneously analyze IgG responses to multiple antigens from multiple pathogens. This study shows the utility of IgG detection by using this assay for monitoring exposure history of a population to an infectious disease and predicting populations most susceptible to exposure after arboviral introduction to a naive population. Because the initial aim of laboratory data collection for this serosurvey was to measure serologic responses to malaria in Haiti, the addition of microbeads coupled to the CHIKV-E1 antigen to the laboratory assay cost <$700 USD, and no additional time was required for data collection. Future serosurveys representative of a population may offer opportunities to collect data on arboviruses and other infectious diseases to provide health officials with quality data to direct disease programs.

Technical Appendix Figures illustrating comparison of anti-chikungunya virus IgG ELISA to bead assay results and anti-CHIKV bead assay IgG signals sampled before and after introduction of CHIKV to Haiti.
