# Supplementary material for: Use of Bead-Based Serologic Assay to Evaluate Chikungunya Virus Epidemic, Haiti
Source: Emerg Infect Dis. 2018 Jun;24(6):995–1001. doi: 10.3201/eid2406.171447 (PMC6004842; doi:10.3201/eid2406.171447)
Supplement: Technical Appendix — Figures illustrating comparison of anti-chikungunya virus IgG ELISA to bead assay results and anti-CHIKV bead assay IgG signals sampled before and after introduction of CHIKV to Haiti. [file 17-1447-Techapp-s1.pdf]

Article DOI: https://doi.org/10.3201/eid2406.171447

Use of Bead-Based Serology Assay to Evaluate Chikungunya Virus Epidemic, Haiti

Technical Appendix

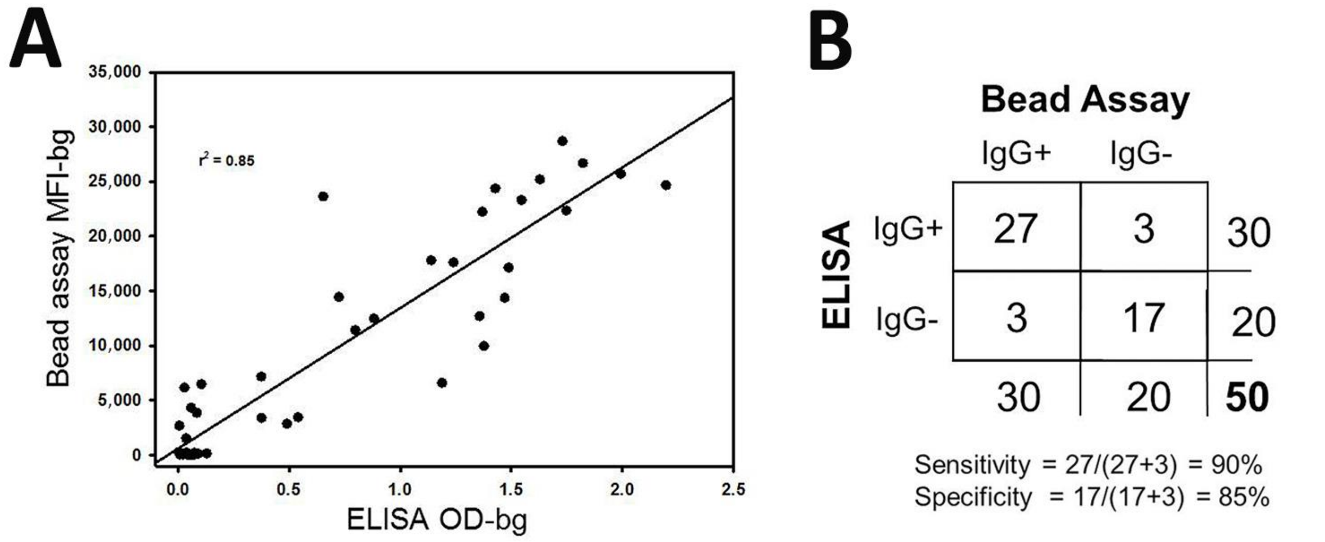

Technical Appendix Figure 1. Direct comparison of chikungunya IgG ELISA and bead assay. A) Scatterplot of assay signal intensities for human serum panel as assayed by ELISA on x-axis versus bead assay on y-axis. Linear regression line shows through data with R^2 value reported. B) Two-by-two table comparing ELISA to bead assay IgG detection results, and estimated sensitivity and specificity if considering the ELISA assay the gold standard test.

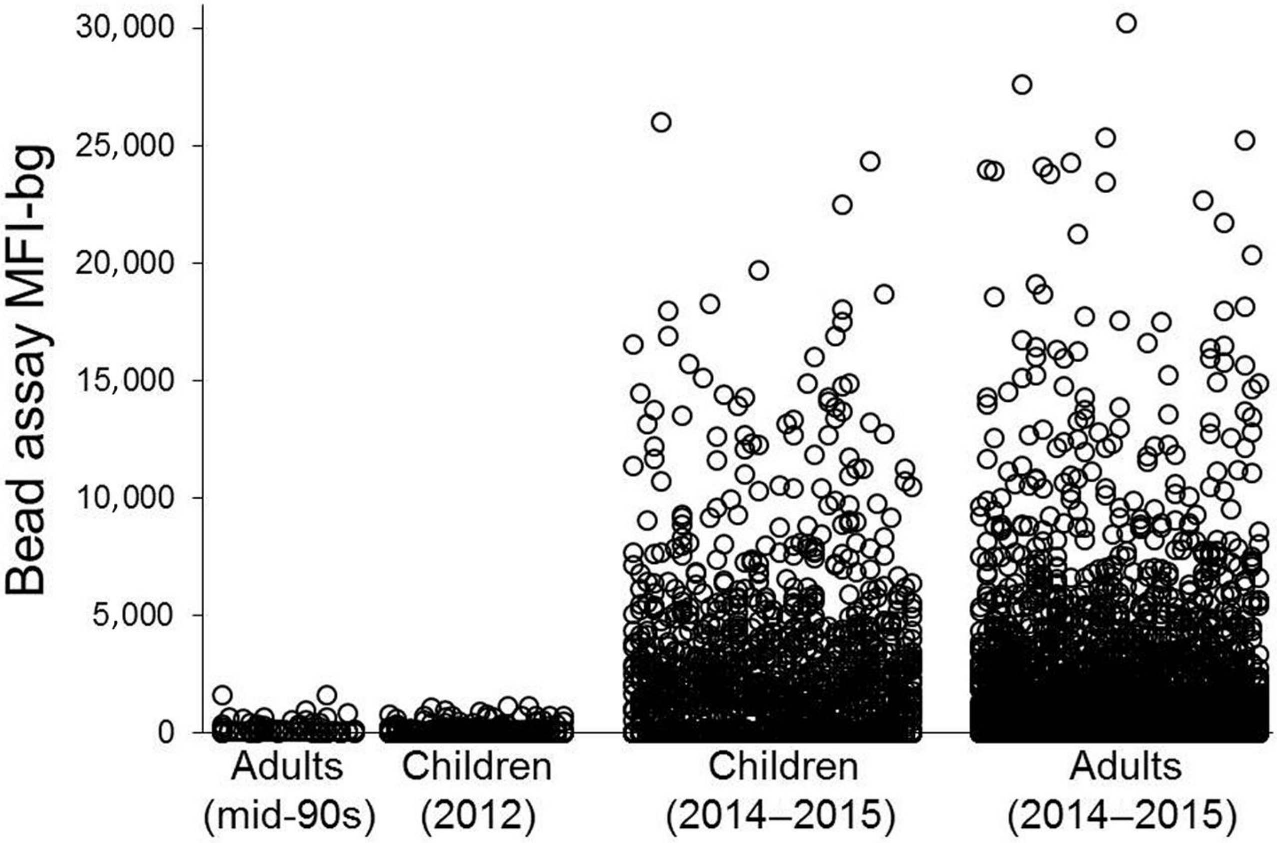

Technical Appendix Figure 2. Comparison of chikungunya (CHIKV) bead assay IgG signals for different Haitians sampled before and after introduction of CHIKV to Haiti. Plot shows anti-CHIKV IgG MFI-bg assay signal for Haitian adults (n = 88) and children (n = 334) pre-2014 introduction, as well as children (n = 1,531) and adults (n = 2,804) post-introduction.
